# A Visual Servoing Scheme for Autonomous Aquaculture Net Pens Inspection Using ROV

**DOI:** 10.3390/s22093525

**Published:** 2022-05-05

**Authors:** Waseem Akram, Alessandro Casavola, Nadir Kapetanović, Nikola Miškovic

**Affiliations:** 1 Department of Informatics, Modeling, Electronics, and Systems (DIMES), University of Calabria, 87036 Rende, Italy; a.casavola@dimes.unical.it; 2 Laboratory for Underwater Systems and Technologies (LABUST), Faculty of Electrical Engineering and Computing, University of Zagreb, 10000 Zagreb, Croatia; nikola.miskovic@fer.hr

**Keywords:** autonomous vehicles, inspection, aquaculture applications, computer vision

## Abstract

Aquaculture net pens inspection and monitoring are important to ensure net stability and fish health in the fish farms. Remotely operated vehicles (ROVs) offer a low-cost and sophisticated solution for the regular inspection of the underwater fish net pens due to their ability of visual sensing and autonomy in a challenging and dynamic aquaculture environment. In this paper, we report the integration of an ROV with a visual servoing scheme for regular inspection and tracking of the net pens. We propose a vision-based positioning scheme that consists of an object detector, a pose generator, and a closed-loop controller. The system employs a modular approach that first utilizes two easily identifiable parallel ropes attached to the net for image processing through traditional computer vision methods. Second, the reference positions of the ROV relative to the net plane are extracted on the basis of a vision triangulation method. Third, a closed-loop control law is employed to instruct the vehicle to traverse from top to bottom along the net plane to inspect its status. The proposed vision-based scheme has been implemented and tested both through simulations and field experiments. The extensive experimental results have allowed the assessment of the performance of the scheme that resulted satisfactorily and can supplement the traditional aquaculture net pens inspection and tracking systems.

## 1. Introduction

Today, fish farming plays a key role in food production, and the number of fish farms is increasing rapidly [1]. Typically, fish farming is carried out in open sea net cages that are natural marine environments. These fish cages are prone to various environmental changes that include biofouling, the growth of organisms such as algae, mussels, hydroids and many more. Furthermore, the water movement also causes net deformation and increased stress on the mooring. These environmental changes may cause harm to the net status and fish health. For example, if netting damage happens, and if it not is discovered in time, the fish escape from the net, decreasing growth performance and food efficiency. Thus, to obtain sustainable fish farming, inspection and maintenance must be performed on a regular and efficient basis [2].

Traditionally, fish net pens inspection and maintenance are carried out by expert divers. However, this method poses high risks to human life and health because of strong oceans waves and currents in the marine environment. A recent trend in the literature is the use of remotely operator vehicles (ROVs) or autonomous underwater vehicles (AUVs) for underwater fish net pens inspection tasks. These vehicles offer low-size and low-cost effective solutions for the aforementioned tasks and can automate the operations using advanced information and communication technology, intelligence control and navigation systems. The use of sonar, compass and depth sensors allows real-time localization without the need for a geographic positioning system (GPS). Furthermore, the use of camera sensors and current computer vision methodologies provides real-time streaming of the environment and interpretation of the captured scenes [3].

In recent years, many researchers have shown increased interest in the development of methods and techniques for autonomous fish net pens inspection using ROVs/AUVs controlled by computer vision approaches. Some of these works are discussed in a later section of this article (see related work Section 2). From our review of the current state of the art, we have seen many research studies that have made use of computer vision technology for addressing the net inspection problem. Currently, these studies cover net damage detection methods including hole detection, biofouling detection, deformation detection, etc. These detection tasks have been performed through traditional computer vision techniques, and also some work has proposed the use of deep-learning methods. However, this area of research is still under development, and there have been considerable research efforts specifically devoted to the integration of control and detection techniques. The current solutions only focus on the detection part, while there are less or very few attempts toward the automatic control and navigation of the vehicle in the net inspection task.

### Main Contribution

This paper demonstrates how a low-cost and camera-equipped ROV, integrated with a topside server on the surface, can be used for auto-inspection and relative positioning in the aquaculture environment. The main objective of this work is to develop a vision-based positioning and control scheme to cut the use of Ultra-Short Baseline (USBL) and to increase vehicle navigation autonomously. In this regard, we first reviewed in-depth the related work dealing with the autonomous vision-based net inspection problem. From our review, we noticed that most of the work only focused on damage detection and its relative position extraction. None of the related work deals with auto-positioning as well as navigation in a uniform and integrated structure.

In this paper, we present an underwater robotic application for automating the net inspection task in underwater fish farms. The strategy consists of an integrated and hybrid modular-based scheme, developed by using existing and well-known frameworks and tools, i.e., ROS, OpenCV, and Python, which is assessed in both virtual and real environments. To enable cost-effective fish net pens inspection, we propose the use of two-parallel ropes attached to the net plane. More specifically, we use traditional computer vision techniques, i.e., SURF (Speeded Up Robust Features) image features and Canny edge detector, for the detection of reference points in the camera images captured in run-time and point triangulation and PnP techniques for vehicle positions relative to the net. Both monocular and stereo imaging techniques were utilized to assess the robustness and correctness of the scheme. In addition, the ROV is directed by using a closed-loop control law to traverse the net from top to bottom along the net plane. As a consequence, we test our methods in simulation as well as in a real environment that illustrates the natural condition of the net installed on a farm. The corresponding results are presented and discussed.

## 2. Related Works

In this section, we review the currently available solutions for the fish net pens tracking and inspection problem. We start by looking in-depth at the available literature, their contributions and what they missed.

In [4], an autonomous net damage detection based on a curve features method was proposed. In this scheme, a bilateral filter, an interclass variance method and a gradient histogram were used in the prepossessing part. Next, the peak cure of the mesh was calculated and the position of the curve was determined for the tracking objective. The detection was performed by determining the characteristic of the net mesh in the image. The results were assessed both with simulations and real field experiments. However, the work only describes the image processing steps for the whole detection of the net, while the vehicle control and guidance aspects have not been addressed.

In [5], the authors proposed a real-time net structure monitoring system. The idea of integrating positioning sensors with a numerical analysis model is examined. The acoustic sensors were installed on the net. Then, because of different ocean currents and waves, the net position differences were calculated at different time steps. The scheme was used to determine the current velocity profiles in the aquaculture environment that cause the net deformation. However, the scheme needs to integrate communication sensors to provide the positions data to the end users.

In [6], the authors proposed a method for the pose, orientation and depth estimation of the vehicle relative to the net. The fast Fourier transform method was used to estimate the depth values from the camera images with known camera parameters. The scheme was tested both in virtual and real environments. However, also in this work, the vehicle control problem is not addressed.

Fish cage dysfunctionalities trigger system loss both from an economic and operational perspective. The bad infrastructure of the net allows fish to escape. To reduce the death rate of the fish, a periodic inspection is required. In this regard, authors of [7] discussed the design of a small-sized autonomous vehicle-based inspection system for underwater fish cages. The scheme offers net hole detection features while the vehicle navigates autonomously during the inspection. The depth estimation is carried out using the OpenCV triangulation method based on the target detection in the camera image. Based on the depth information, the vehicle is instructed to move forward/backward. The scheme was tested successfully in a real environment. However, to achieve more autonomy in the system, top-down movement control is required.

ROV/AUV-based aquaculture inspection poses localization issues as the GPS system does not work underwater. Alternatively, the surface vehicle area is easy to deploy and maintain with fewer limitations on communication and localization. In [8], authors discussed the design and implementation of an omnidirectional surface vehicle (OSV) for fish cage inspection tasks. A depth-adjustable camera was installed with the vehicle that captures the net structure at different depths. Furthermore, the net damage detection problem was solved by using a pretrained deep-learning-based method. However, the factors that interfere with the position estimation were not incorporated. In [9], authors presented an extension of a former work by incorporating the artificial-intelligence-based mission planning technique. A hierarchical task network was exploited to determine the rules for vehicle movement. However, the scheme is not validated in a realistic environment.

The traditional methods for biofouling removal are costly and have a great impact on fish net stability and fish health. The waste products are left in the water creating a bad environment for the fish. In this regard, ROV/AUV-based biofouling detection and removal provide a more sophisticated solution. In addition, static sensors are also used to regularly monitor environmental conditions. Authors of [10] reported a detailed theoretical analysis of robotic solutions for biofouling prevention and inspection in fish farms. Various technical and operational requirements are proposed and discussed. The study proposed an automatic robotic system for biofouling detection and cleaning that consists of environmental condition monitoring, net and biofouling inspection, growth prevention and fish monitoring inside the cages. As a result, that work proposed specifications and requirements for the development of such a system that offers detailed guidelines for the deployment of the robotic system for the aquaculture inspection task.

In [11], the authors proposed a novel method for net inspection problems. This work suggested the use of a Doppler velocity log (DVL) to approximate the vehicle’s relative position in front of the local region of the net. The position coordinates are then used in line-of-sight guidance control laws for the heading movement at a constant depth and angle with respect to the net plan. However, the scheme required noise handling in DVL to achieve better tracking results. Furthermore, due to an unfriendly environment, a more robust control law is required to deal with the model uncertainty.

In fish farming, water quality matters for the health of cultured fish. Thus, water quality assessment is also an issue of great interest in the fish farming environment. In [12], the authors presented a fish cage inspection system that integrates monitoring water quality along with the net status. Different sensors were installed on the net to monitor potential hydrogen (pH), oxidation reduction potential (ORP), dissolved oxygen (DO) and temperature. For net damage detection, a Hough Transform method was used to construct the net mesh, and based on the incomplete net pattern, the damaged part was detected in the camera image. Although the work was tested in an experimental environment, the vehicle was controlled manually. Similarly, the authors in [13] deployed hardware and software solutions including SeaModem for communication, HydroLab for water quality monitoring, and energy harvesting system through propellers in underwater fish farms via acoustic IoT networks.

Another regular inspection of the fish cage net is carried out in [14]. In this work, the distance control scheme is presented for net status inspection through video streaming in a real-time environment. This scheme requires a physical object attached to the net considered as the target location. Then, computer vision methods, e.g., canny edge detector, are used to detect the target in the image under fixed distance and angle. The target information is then used to instruct the vehicle to move forward/backward toward the net plan. Although the presented scheme is simple and easy to deploy, it requires that predetermined target objects be attached to the net surface. Additionally, the controller is not robust to environmental disturbances and noise.

Traditional positioning methods involving the use of a long-baseline and ultra-short baseline methods, require predeployed and localized infrastructure, increasing the cost and operational complexity. On the other hand, the laser and optical systems are easy to deploy and are efficient solutions in a dynamic environment. In this regard, the authors of [15] proposed a laser–camera triangulation-based autonomous inspection of the fish cage net. In this scheme, the idea is to project two parallel laser lines on the net plan. By using image processing techniques, the lines were extracted from the images and their positions were estimated by the triangulation method. This approach showed better results when compared to the DVL method. However, this work only suggested the position estimation for the net tracking problem and does not consider the underlying control problem. The laser triangulation method needs to be used in a closed-loop under a tracking controller suitably designed for tracking purposes.

A trend in recent years is to introduce artificial intelligence and Internet of Things technology in aquaculture systems to obtain real-time information and optimal aquaculture performance. In [16], an attempt has been made toward the application of an IoT-based smart fish net cage system. In this work, the authors developed a smart cage system that integrates artificial intelligence, IoT, a cloud system, big data analysis, sensors and communication technology. The system communicates field information to the cloud where the big data analysis is performed. The system generates real-time information related to fish health, survival rate and food residuals. However, this work only considered data collection and processing. Vehicle autonomous control and guidance problems are not considered.

Fish cages are floating structures, and it is difficult to get a planned image of the net through camera imaging. More robust image processing is required to deal with the different net structures, shapes and sizes. The different blurred scenes should also be considered. In this regard, the authors in [17] studied net hole detection of different shapes and sizes under different underwater conditions. In this work, a combination of Hough Transform and statistical analysis methods were used to perform a local and global search for detection problems. The work was only tested on the offline images sequence. However, the work needs to be tested on a real vehicle in real-time systems to verify its relevance.

Recent studies in [18,19,20] discussed the development and results of the HECTOR- heterogeneous autonomous robotic system in viticulture and mariculture project. The purpose of the project is to use an unmanned aerial vehicle (UAV), unmanned surface vehicle (USV) and ROV in an integrated and coordinated manner to carry out different missions such as vineyard surveillance, spraying and bud-rubbing in the viticulture domain and fish net monitoring in the mariculture domain. The research carried out as a part of the HECTOR project in [21] developed an autonomous control scheme that allows vehicles to navigate autonomously while performing the detection of net status. In this work, an ROV was allowed to move autonomously and stream video to the topside computer, perform image processing to detect two parallel ropes in an image considered as target positions and then generate the velocity commands to the vehicle for implementing a distance and top/down control. Additionally, a pretrained deep neural network was used to perform real-time biofouling detection on the net. However, the proposed scheme is not robust in the sunlight and produces blurred images in a real-time environment.

In the literature, pose estimation is mainly performed with feature-matching techniques. However, such approaches are prone to generate inconsistent results in the estimation because of the similarity in different regions of the net plan. To overcome this problem, the authors in [22] proposed a novel pose estimation method by considering junction detection. In this work, the knots of the net and their topology in the camera image were used in the pose estimation relative to the camera position. This approach cuts the computation burden of feature extraction from the image on the system performance. However, vehicle control and localization are not discussed. Moreover, the pose estimation is not robust in distorted images.

As we have seen, ROVs are mostly used for the autonomous fish cage inspection problem. However, ROVs feature low maneuverability and low efficiency in limited working space and a long and dynamic environment. To improve the performance of ROVs in inspection tasks, the authors of [23] developed a novel inspection scheme called Sea Farm inspector. The system integrates a ROV with a surface vehicle for the fish net inspection and tracking problem. The surface vehicle is responsible for controlling and communicating with the ROV during the operations. Furthermore, the design and control scheme is described in the work. However, this work is at the initial phase and the real implementation is ongoing. Further extensions have been undertaken in the work (17). In the latter study, the authors provide system coherence by properly integrating a surface vehicle, winch and ROV. However, the camera integration for net inspection is still being developed.

Next, a summary of the reviewed related work is shown in Table 1.

## 3. Design, Algorithm and Implementation

In this section, we present the design and implementation details of the proposed vision-based control scheme for the net inspection problem.

### 3.1. Overview

The proposed scheme follows a modular approach consisting of two modules. The first module is responsible for distance estimation using traditional computer vision techniques. The second module is responsible to guide and control the vehicle movement along the net plane to inspect its status. In this work, two different designs are investigated, namely Method 1 and Method 2, based on the camera use. There are a certain number of other applications that can profit from the proposed visual control strategies, see, e.g., [24,25,26].

In the following, a detailed description of the system is provided.

### 3.2. Distance Estimation

In this section, we describe distance detection by considering a reference point in the image frame of the net. We employed two different methods that are described hereafter:

#### 3.2.1. Method 1: Stereo Image Design

In this section, we discuss the design of the proposed Method 1. The proposed idea is taken from [27] and further elaborated in this work. In the latter, a vision-based positioning system is presented for docking operation of the USVs in the harbor environment. We extend the solution of that previous work by allowing the online detection of the target positions in images, and the generated path is used to solve a fish net tracking problem for an ROV model.

**Figure 1:**
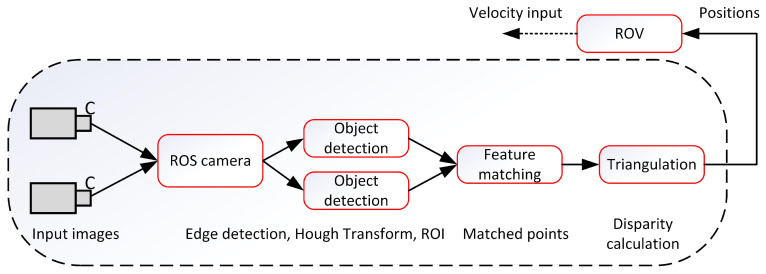
Method 1. A stereo image design scheme for positioning and auto-inspection of the underwater fish cage net adopted from [27].

In Figure 1, a schematic of Method 1 is shown. The forward-looking (left and right) cameras installed on the vehicle are used to collect the image sequences. The “cv-bridge” package is used to convert the obtained images from ROS to OpenCV formats. Next, the obtained images are forwarded to the object detector that extracts the net and draws a bounding box around the region of interest (ROI) using a canny edge detection algorithm. Next, from the right image, the image SURF features are extracted from the bounding box and searched in the corresponding bounding box of the left image along the horizontal lines. The matched points are used to compute the disparity map based on the triangulation method. Finally, the disparity map is used to obtain the relative positions of the vehicle with the objective to traverse the net from top to bottom while keeping a safe distance from it.

**Figure 2:**
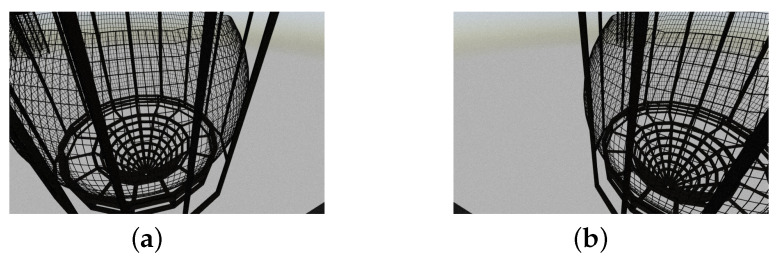
Input images from the vehicle cameras: (**a**) left camera’s image; (**b**) right camera’s image.

In this method, a stereo imaging design is employed. First, two raw images from the available cameras are collected as shown in Figure 2.

The obtained images may contain some noise and distortion. Then, we recover the rectified images by using the “unDistortRectify” OpenCV method. This requires the availability of the camera matrix and distortion coefficients obtained from the calibrated camera installed along with the simulator.

(1) $$A=\left[\begin{array}{ccc} fx & 0 & cx\\ 0 & fy & cy\\ 0 & 0 & 1 \end{array}\right]$$

where fx and fy are the focal lengths, and cx and cy are principal points. Moreover,

(2) $$K=[k_{1},k_{2},k_{3},p_{1},p_{2}]$$

are the distortion parameters, where $k_{1},k_{2},k_{3}$ denote the radial distortion parameters and $p_{1},p_{2}$ the tangential ones [15].

**Figure 3:**
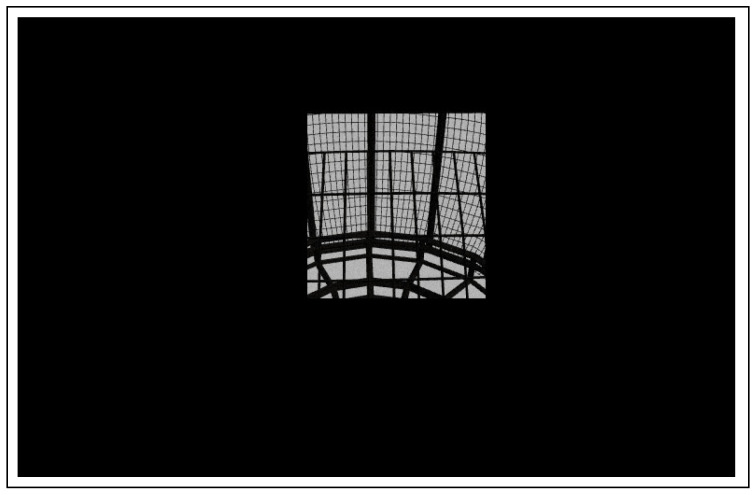
This figure illustrates how ROI is extracted. This region is used for edge detection and features extraction.

Next, a small region of interest (ROI) is selected in the image. Because the whole net has the same pattern, it is easy to select a small portion of the net to reduce the computational burden during the features extraction. The selected region of interest of size “300 by 200” pixel is shown in Figure 3.

**Figure 4:**
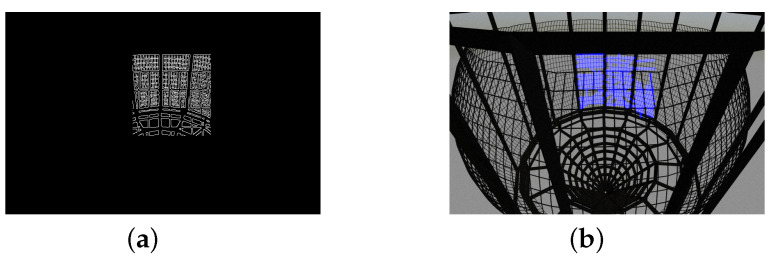
Output images after applying Canny edge detector: (**a**) detected edges in ROI; (**b**) resulted image.

Next, we can recover the edges in the ROI image by calling up the “Canny” edge detection algorithm. The Canny algorithm is a popular mathematical operator that finds prominent edges in images using a multi-stage approach consisting of noise reduction, finding intensities, non-maximum suppression and thresholding. This step is required to check if there are enough pixels related to the net area inside the selected ROI. Otherwise, the search and extraction of the features of interest may not be effective. The final result containing strong edges in the image is shown in Figure 4.

**Figure 5:**
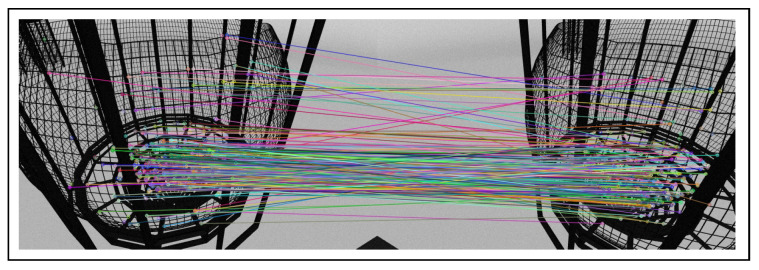
Matched points in both right and left image.

The next step is to design the stereo vision system. Here, the epipolar geometry concept is employed. The basic idea of the system is to search the SURF features in the bounding box containing the detected edges in the right image. SURF features are a scale and rotation-invariant interest point detector and descriptor [28]. Next, the same feature extraction steps are followed in the bounding box by considering horizontal scale lines corresponding to the left image. The next phase is to perform feature matching and find the best match in the corresponding left and right images. For points matching purposes, the K-Nearest Neighbour routine by OpenCV is used, and a filter is applied to detect only the best matches. Those are the ones that have a constant distance less than 0.6, and they are labeled as a best-matched point. Finally, the best match points are obtained and drawn as shown in Figure 5.

Algorithm 1 returns the pixel positions of best-matched points in the left and right images that are further sorted according to the distance value, and the points pair with minimum distance is selected which is used to calculate the pixel difference of these two points to compute the disparity. Then, the distance value is obtained with the help of the following formulas:

(3) $$\begin{array}{c} f=(w/2)/(\tan(fov/2))\\ d=|cxl-cxr|\\ distance=(f*b)/(d)\\ x=((cxl-cx)*b)/(d)\\ y=((cyl-cy)*b)/(d) \end{array}$$

where *f* denotes the focal length, *w* denotes the width of the image frame, fov the camera angle, *d* the disparity, cxl and cxr the pixel coordinates of the points in the left and right images, cx and cy the center midpoints of the image, and *b* the baseline, that is the distance between the two cameras.
**Algorithm 1** Stereo vision-based positioning algorithmInitialization:1:**set:** camera-left, camera-rightOnline Phase1:**for**t>0**do**2:    **get-images:** Read images from both cameras3:    **do-rectify:** Remove distortion4:    **get-roi:** Select region of interest (ROI)5:    **get-edges:** Apply canny edge detector6:    **draw-contours:** Calculate a rectangular region containing the detected edges7:    **find-features:** Extract features present in contours8:    **match-features:** Match features pairs in the second image9:    **filter-matched-features:** Apply filter to get the best match feature pairs10:  **return** the pixel positions of the matched best feature pair11:**end for**

#### 3.2.2. Method 2: Monocular Image Design

In this section, we discuss the design of the proposed Method 2 that is achieved by elaborating and extending ideas presented in [21]. Specifically, we have generalized the approach for the Blueye Pro ROV in a field environment and performed the assessment of the performance of the scheme. The basic idea of the scheme here is to identify two parallel ropes attached on the net in the image frame and then determine the position of the rope in the image to perform the distance estimation of the net with respect to the vehicle.

**Figure 6:**
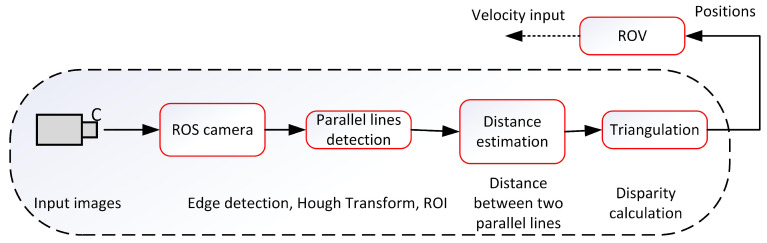
Method 2. Monocular image design scheme for positioning and auto-inspection of the underwater fish cage net adopted from [21].

In Figure 6, a schematic of Method 2 is depicted. Both methods share the same functionality except for the usage of the cameras. In Method 2, a monocular camera is used. Here, the idea is having two parallel ropes along the net surface. Then, by means of edge detection and Hough transform algorithms, the ropes in the image are extracted, and pixel distance is calculated. Next, by using the computed pixel distance and knowing the real distance between the ropes, the positions are obtained which are the necessary input to the vehicle control and navigation algorithms.

**Figure 7:**
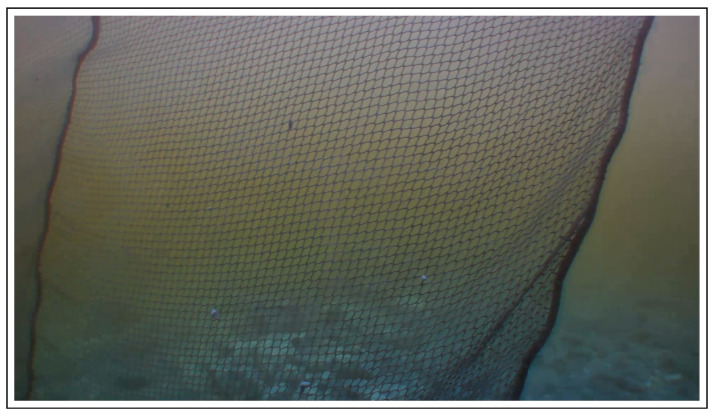
An example of the input image from the blueye vehicle camera obtained during the experiments.

The vehicle on-board camera is used to capture the cage net as shown in Figure 7. The input image is of size “1920 × 1080”—a high resolution image. To make the detection process easier and robust, it is necessary to undertake some preprocessing steps. Thus, first we modify the input image by applying the “CLAHE” OpenCV method. The CLAHE (contrast limited adaptive histogram equalization) recalculates the values of each pixel in the image and redistributes the brightness level, thereby increasing contrast in the image. This results in better visibility of the image objects and makes the identification easier. Next, the image is converted to gray and the “Bilateral filter” by OpenCV is used. The filter makes use of one or more Gaussian filters and blurs the neighborhood pixels of similar intensities while preserving the edges. The image dimension is reduced by a 25% of the original one with the intention to eliminate the unnecessary details in the image. The resulting image is then used for the distance estimation process.

**Figure 8:**
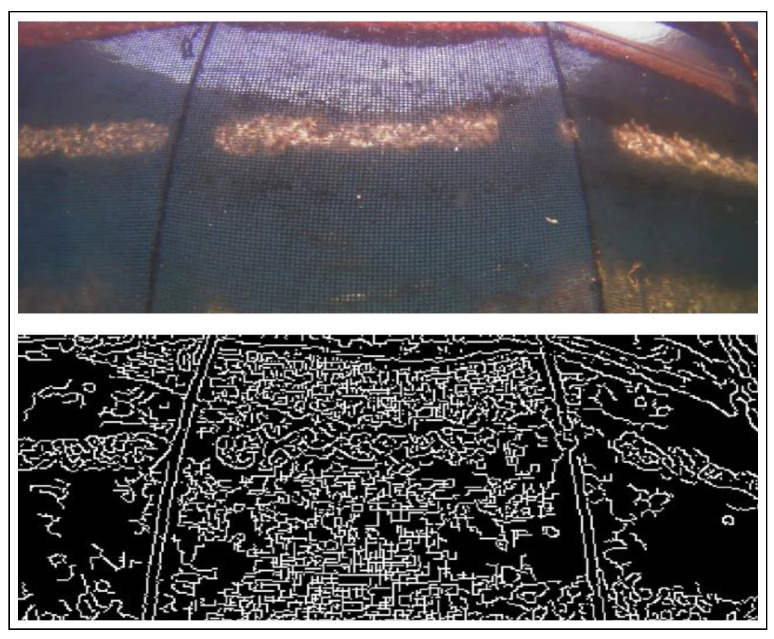
Results after preprocessing the original image and applying the Canny edge detector.

The next essential step is the identification of the two parallel ropes in the image. The ropes are recovered by applying the Canny edge detection algorithm. The algorithm follows a three step-procedure which include noise reduction, the calculation of the intensity gradient, the suppression of false edges and hysteresis thresholding. The resultinb image is shown in Figure 8.

The ropes in the image can essentially be considered as parallel straight lines. As we are only interested in these lines, we can freely discard the minor edges and only extract the large edges in the image. Therefore, the detection of the lines is achieved by applying the Hough transform method. This method requires an input image containing all edge information obtained from the previous steps and uses gradient information for the detection of the lines. The gradient is a measurement of the changing intensities of pixel coordinates inside an image and mathematically can be written as:

(4) $$\nabla f\left(x,y\right)=\left[\begin{array}{c} G_{x}\\ G_{y} \end{array}\right]=\left[\begin{array}{c} \partial f/x\\ \partial f/y \end{array}\right]$$

where $G_{x}$ is the gradient of the x-axis, $G_{y}$ is the gradient of the y-axis, ∂f/x shows change in intensity of the x-axis, and ∂f/y shows change in intensity of the y-axis. Furthermore, the size and direction of the gradient are calculated by:

(5) $$\begin{array}{c} \left|\nabla f\right|=\sqrt{G_{x}^{2}+G_{x}^{2}}\\ \theta =\arctan G_{y}/G_{x} \end{array}$$

Given the size (|∇f|) and direction (θ) of the gradient, the direction of an edge is determined by a perpendicular line at any given point in the image. Next, to draw the lines in the image, the polar coordinate system is used

(6) r=xcos(θ)+ysin(θ)

**Figure 9:**
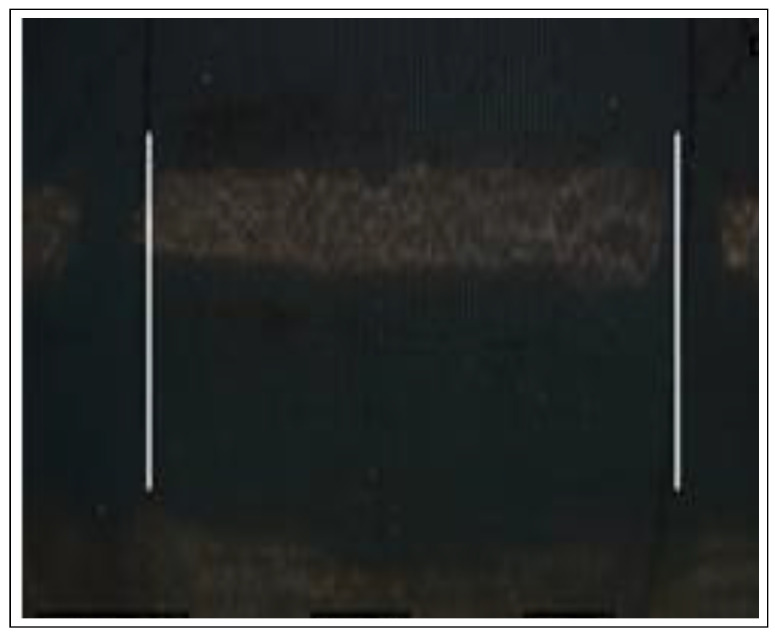
Output image with two identified parallel lines after applying OpenCv methods.

The pair (r,θ) shows the intersection point of a line that passes through two points (x) and (y). Here, *r* denotes the distance from the origin to the nearest point on the line, and θ denotes the angle between the x-axis and the line which connects the origin with that nearest point. Thus, each line in the image is constructed, and the resulting image is shown in Figure 9.

The overall procedure of Method 2 is summarized in the following algorithm.

Algorithm 2 returns pixel positions of the detected two parallel lines in the image based on pixel differences and is calculated by:

(7) $$\begin{array}{c} d=|PL-PR|\\ distance=(f*object/d)*scale \end{array}$$

where the term *d* denotes the difference between average pixel values detected in left and right ropes in the images that are denoted by PL and PR, respectively, and the object denotes the real distance between the two ropes that need to be known in advance, and the term scale takes into account the ROV camera tilt angle.
**Algorithm 2** Monocular vision-based positioning algorithmInitialization:1:**set:** cameraOnline Phase1:**for **t>0**do**2:    **get-images:** Read image from camera3:    **pre-process:** Improve image quality4:    **get-edges:** Apply canny edge detector5:    **draw-lines:** Apply Hough-lines transform algorithm6:    **separate-lines:** Get the two parallel lines7:    **return** the pixel positions of the obtained parallel lines8:**end for**

### 3.3. Control Law

The ROV is described in 4 DOFs: surge, sway, heave and yaw. To solve the control design problem, first we assumed that:

- The roll and pitch motion is passively stabilized by gravity and can therefore be neglected.
- The vehicle is neutrally buoyant, and the motion in heave can therefore be neglected.

In particular, here we focus on the control of surge, sway, heave and yaw speed of the vehicle to perform the net pens inspection task. The control law is designed which directs the ROV heading toward the net pen and makes the ROV traverse the net pen with a desired distance and speed. This way, the camera view is directed toward the net such that the ROI stays in camera view while the ROV is traversing.

The control module is used to instruct the vehicle to perform a predetermined course outside the net to generate live streaming to the topside user and to inspect its status [21]. This part complements the methodology of the auto-inspection of nets using ROVs. The control commands use the position and distance information obtained from the object detection module via computer vision methods on the acquired images of the vehicle cameras. To this end, a simple but efficient control law is synthesized that generates the velocity set point for vehicle movement based on the reference position and distance data. The overall navigation problem can be stated as follows:

Given the depth information, generate the velocity commands to move the vehicle from top to bottom under a certain predefined distance while keeping the net plane in a parallel position with respect to the vehicle position.

In view of the above statement, the algorithm works as follows. First, the distance estimation module is called that preprocesses and detects target points in the images to identify the reference point. It checks if the target is visible or not. If the target is not visible, a rotation command is sent to the vehicle. Once the target is detected, it checks if the distance between the vehicle and the net is in the range of the predefined distances. If too far or too close, the forward/backward commands are sent to the vehicle, respectfully. Once the vehicle is at the desired distance, a top to bottom movement command is sent to the vehicle until the bottom area is detecte, and the navigation is stopped. The overall control procedures are explained with the help of Algorithm 3.

The goal of this study is the design of a control method allowing the ROV to traverse the net pens from top to bottom. Once the ROV reaches the bottom of the net pens, the distance estimation module is not receiving the input and sends a stop command to the vehicle. Once the one drive is completed, ROV is manually lifted to the docking/surface position. Incorporating the autonomous docking capability, in addition to the proper control of the heading and velocity, is out of the paper scope and will be addressed in a future study.
**Algorithm 3** Control and Navigation algorithmInitialization:1:**set:** target positions2:**choose:** ref-distance3:**store:** net-distance, wanted-distance, x-force, z-forceOnline Phase1:**for **t>0**do**2:    **compute:** the net-distance by solving (3) or (7)3:    **if** net-distance > ref-distance **then**4:        **move-fwd**5:    **else if** net-distance < ref-distance **then**6:        **move-bwd**7:    **else if** net-distance==ref-distance **then**8:        **move-dwn**9:    **else**10:        **wait**11:    **end if**12:**end for**

## 4. Results

The proposed schemes used, both in simulations and experiments, a ROV and net pens with the same characteristics. First, the structure of the net pen was developed in the blender tool that supposedly roughly covered the size of the net pens used in the experiments. In addition, the methods only need to know in advance the distance between the reference points attached to the net pens. The ROV was used for image acquisition and tracking purposes, which is evident both from the undertaken simulations and experiments.

Following the design of the proposed vision-based control scheme, we move over to the results. The results are divided into two parts, i.e., Simulation and Experiments, where each focuses on the different design choices proposed in this work. Here, we discuss how the experiments were conducted and what we achieved. Finally, based on the obtained results, we make some conclusions.

### 4.1. Simulation Results

#### 4.1.1. Description

To test the proposed Method 1 and Method 2 schemes in the simulation setting, we adopted the “unmanned underwater vehicle simulator” (UUV) simulator [29]. It is a set of packages that includes plugins and ROS applications that allow carrying out simulations of underwater vehicles in Gazebo and Rviz tools.

The simulation environment consisted of an underwater scene and the ROV vehicle that performed the tracking tasks. To simulate the net-tracking scenario, we designed the net structure using the blender tool. The simulation was performed on ROS Melodic distribution installed on Ubuntu 18.04.4 LTS.

In the simulator, the vehicle model named “rexrov-default” was used that consists of a mechanical based with two cameras and other sensing devices, e.g., inertial measurement unit (IMU) and LIDAR. Furthermore, the Doppler velocity log (DVL) was also available to measure the vehicle velocity during the simulation. The model also consisted of the implementation of Fossen’s motion equations for the vehicle [30] and the thruster management package.

**Figure 10:**
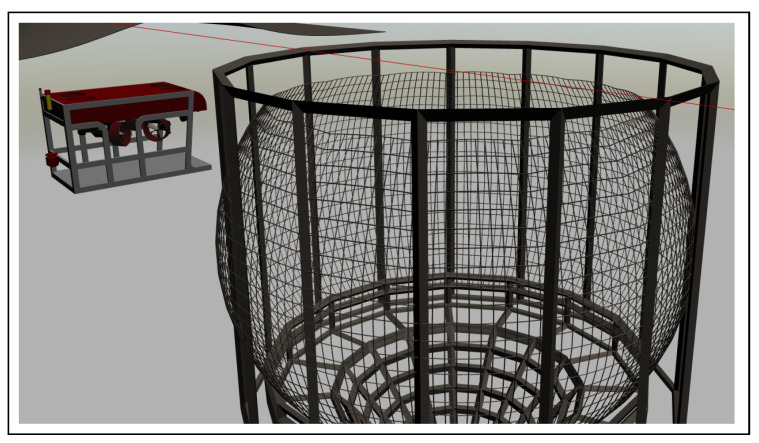
Simulation setup. Model initialized in Gazebo simulator.

By following the stereo and monocular imaging design, the two forward-looking cameras were used during images acquisition with the objective of testing the vision-based auto-inspection of the net structure. The simulation setup is shown in Figure 10 where the vehicle model is cloned in the underwater scene and facing toward the net plane.

#### 4.1.2. Results

**Figure 11:**
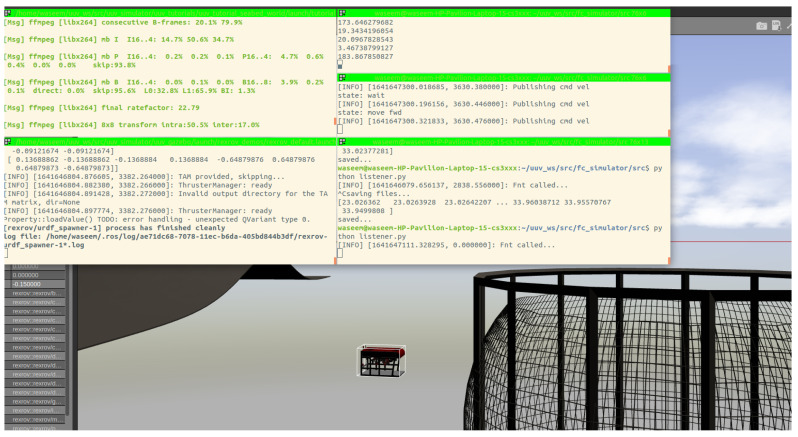
Simulation screen captured during run-time.

In this section, the simulation results are described. The main objective of the simulation was to test the performance of both Method 1 and Method 2 described in the earlier sections for distance estimation and tracking purposes. First, as an example, the working of the scheme is shown in Figure 11 where different ROS nodes are called in the terminal windows and communicate with the vehicle.

**Figure 12:**
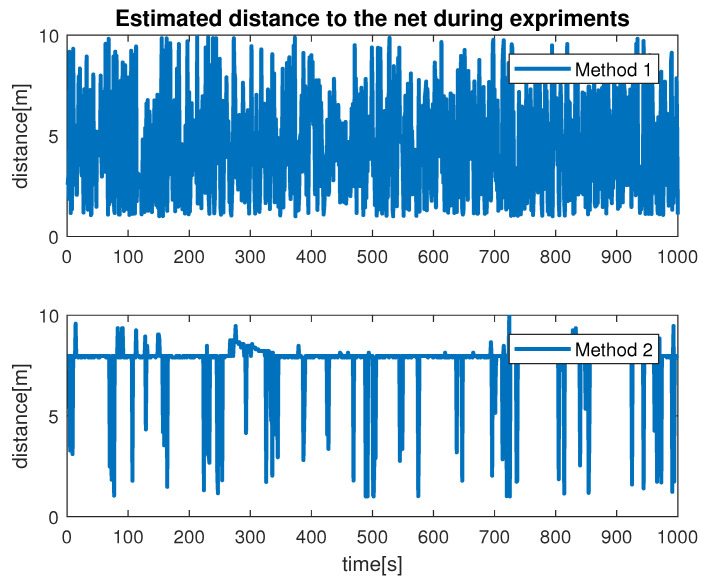
Fish cage net distance estimation during simulation.

**Figure 13:**
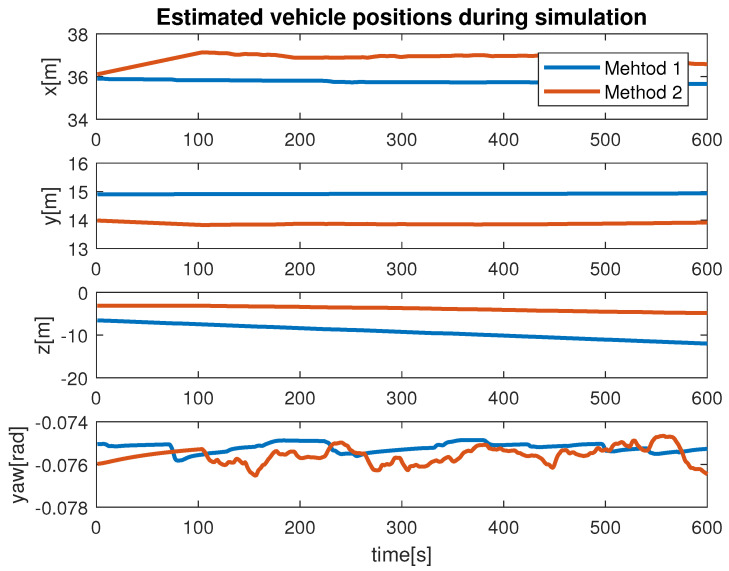
Vehicle positions during simulation.

**Figure 14:**
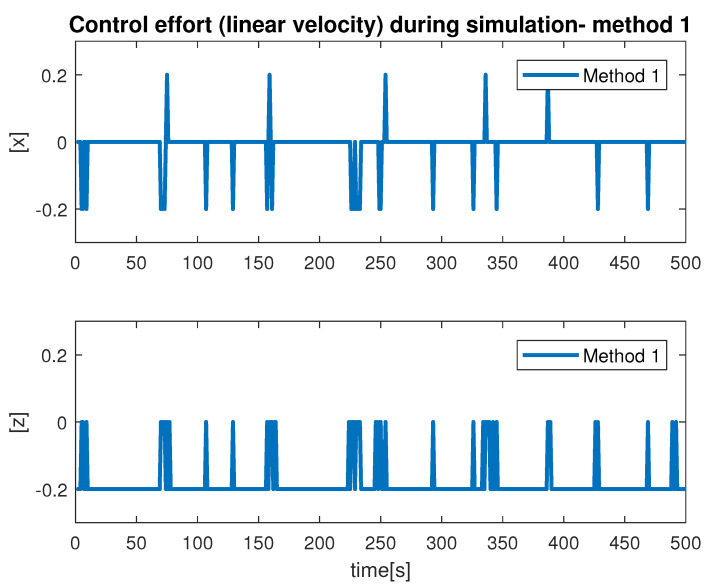
Velocity profile by Method 1.

**Figure 15:**
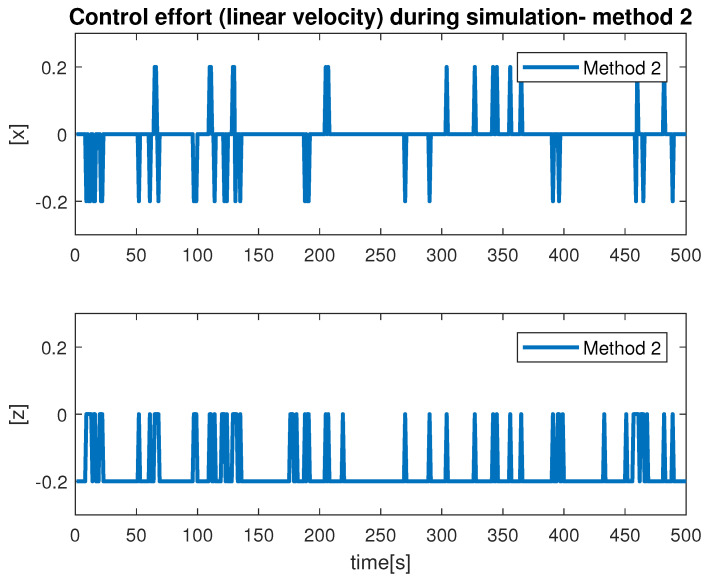
Velocity profile by Method 2.

During the simulation, we observed the estimation of the distance between the vehicle camera and the net plane via Method 1 and Method 2. This is shown in Figure 12. From our results, we found that the monocular image-based method produced smooth results compared with the stereo-image-based method. The signal produced by Method 1 had multiple abrupt changes during the simulation. This was due to the fact that if the obtained images were blurred or not flat, the scheme extract features incorrectly caused the ambiguous estimation. From our experiments, we learned that Method 1 is highly influenced by the choice of hardware, feature extraction and computation cost. In contrast, Method 2 showed reasonable performance for the distance estimation. We can conclude that Method 2, which makes use of the monocular imaging design technique, is feasible for tracking underwater fish net pens at sea. The second test that we performed was finalized to observe the vehicle states during the simulation. This is shown in Figure 13, where the vehicle positions (x, y, z and yaw) achieved by applying Method 1 and Method 2 can be seen. In terms of performance, we noticed that Method 2 showed better performance compared to Method 1. This can be seen more clearly in the state *x* and angle yaw. With Method 1, the path covered was not linear despite the absence of external noise imposed in the control path. Furthermore, the state *z* confirmed the top/down traversing during the simulation. From our results, we can conclude that Method 2 is more suitable to be used for tracking. The third test was organized to analyze the velocity profiles during the simulation. This is shown in Figure 14 and Figure 15. Here, the results show the velocity set points for x and z. The x set point was used for the longitudinal movement while the z set poin twas used for the depth movement of the vehicle. The results confirm that whenever the vehicle stays at the desired distance from the net, the controller generates the z velocity set point correctly. From our analysis, we can conclude that the proposed schemes could be used to address the tracking problem.

### 4.2. Experimental Results

#### 4.2.1. Description

**Figure 16:**
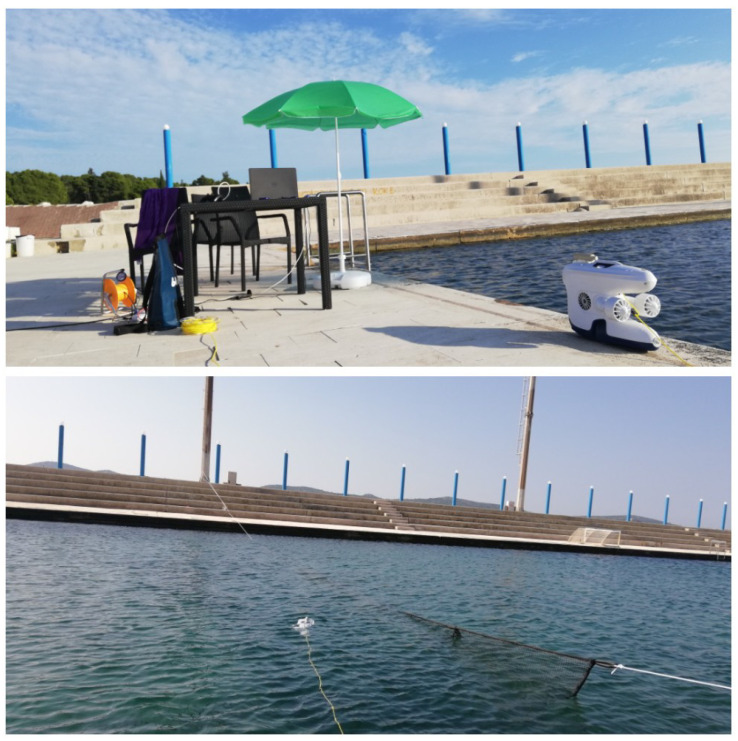
Field trial view before experiments.

To test the proposed Method 2 scheme in a real environment setting, a small set of experiments were conducted during the workshop “BTS (Breaking the Surface) 2022” organized by the Laboratory for Underwater Systems and Technologies (LABUST) at the Faculty of Electrical Engineering and Computing, University of Zagreb in October 2021 at Biograde de Muro, Croatia. The experiments were performed in the pool setup as shown in Figure 16.

**Figure 17:**
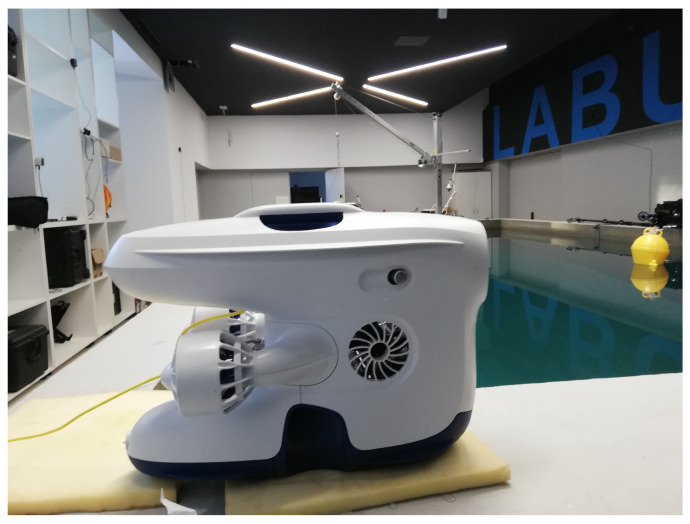
The Blueye Pro ROV.

Blueye Pro ROV as shown in Figure 17 was acquired from the LABUST during the experiments. The Blueye Pro ROV is produced by Blueye Robotics and has dimensions of 48.5 × 25.7 × 35.4 cm length, width and height, respectively. The ROV weighs 9 kg, it can go down to 300 m depth, and it has a 400 m cable for tethering. It is also equipped with a battery allowing 2 h of autonomy. The ROV can be used in saltwater, brackish, or freshwater. The drone moves with help of the available four thrusters of 350 w.

The ROV has a full HD forward-looking camera with a [−30 deg, 30 deg] tilt angle and 25–30 fps imaging capability. Additionally, it is equipped with a powerful light that ensures well-lit imagery under low-light scenarios. Other sensory devices including IMU, accelerometer, compass and temperature are also installed on it. The ROV allows control and communication with a topside user on the surface through both WiFi and Ethernet cable in a wireless environment [18]. Furthermore, the ROV is integrated with an open-source BlueyeSDK-ROS2 interface (see [31]). This allowed for achieving a connection between the Python-based ROS nodes running on a topside computer and the ROV.

#### 4.2.2. Results

In this section, the experimental results are described. The main objective of the experiments was to test the performance of the proposed vision-based positioning and control scheme for the underwater fish net pens inspection problem using the Blueye Pro ROV. Here, we performed experiments by following the proposed Method 2 that makes use of the monocular imaging-based design technique.

**Figure 18:**
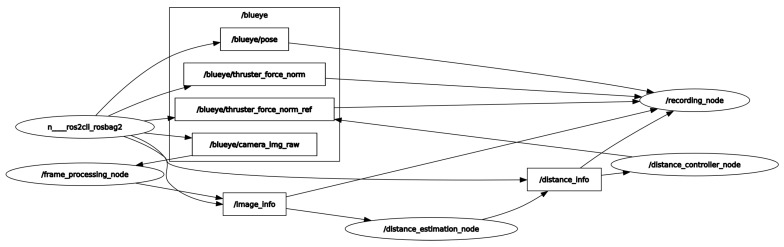
Screenshot of rqt-graph of all the ROS nodes during run-time.

Generally, the adopted ROV had several challenges during the experiments. First, there is no interface provided by the manufacturer where one can get access to the vehicle’s internal parameters which are necessary for the feedback control. Second, there is a lack of vehicle position, velocity and depth estimation. The only possible way to interact with the vehicle is to use the Blueye Python SDK that comes with the vehicle. SDK allowed us to subscribe to the “Thruster force” topic and publish a constant velocity set point to the surge, sway, heave and yaw motion of the vehicle as shown in Figure 18. Another main problem faced during the experiments was the uncalibrated vehicle camera that generated noisy images resulting in degradation of the algorithm performance.

**Figure 19:**
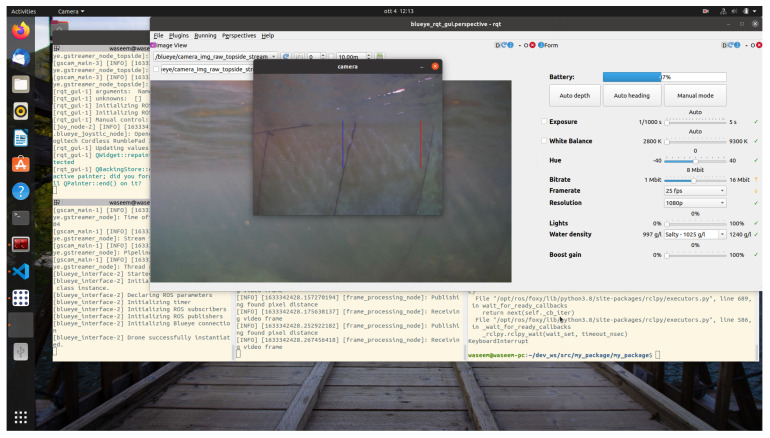
Experiments screen captured during run-time.

**Figure 20:**
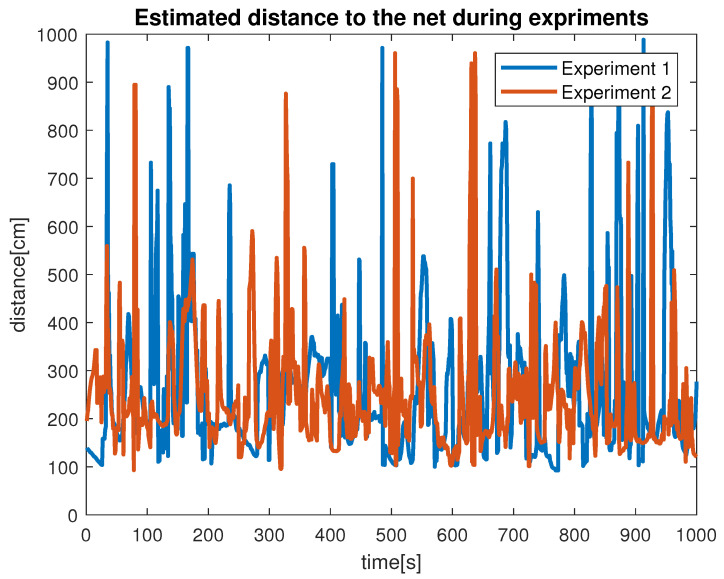
Fish cage net distance estimation.

**Figure 21:**
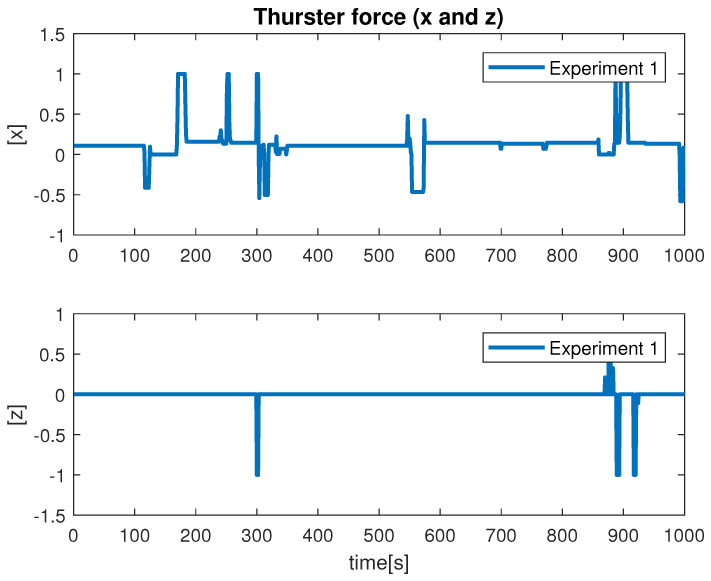
Velocity profile during Experiment 1.

**Figure 22:**
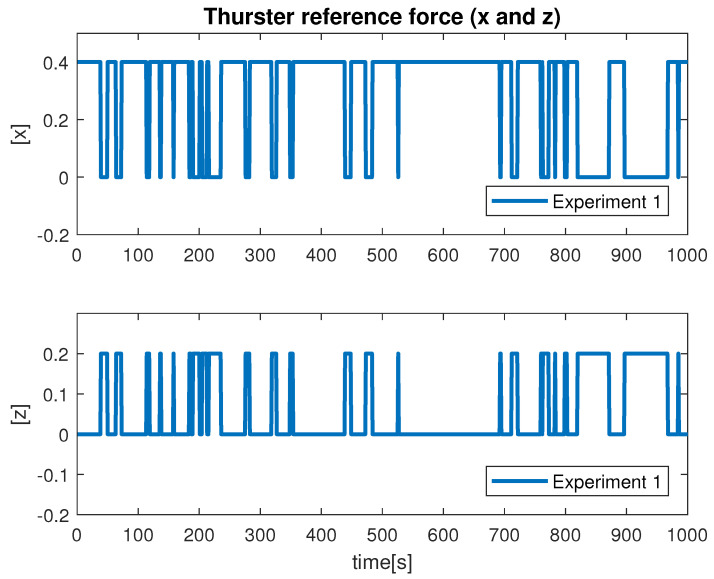
Velocity profile during Experiment 1.

Despite the above-mentioned challenges, the vehicle was used for the distance estimation of the vehicle relative to the fish net pens in a field trial as shown in Figure 16, and some results were collected. In Figure 19 the running interface is shown. In the figure, one can clearly see that the algorithm is working by identifying the ropes as two parallel lines in the input image. Next, these lines are used to estimate the distance to the fish net pens. The estimated distance is shown in Figure 20. The reference distance used during the experiments was 200 cm, and based on the current distance, the vehicle was instructed to move forward/backward. While the vehicle got in range of the wanted distance, the top to down motion was called. Here, we performed two different experiments and examined the estimated distance. The results show similar performance from both experiments. However, false estimation was also observed during the experiments. By clearly examining the results and after tuning the algorithm parameters, we concluded that the estimation process is influenced by the input data. From the experiments, we learned that the algorithm was showing performance degradation with sunlight and poor weather. Moreover, the distance estimation data showed that the proposed scheme was capableof being used for the distance estimation and tracking of the fish cage net. For thoroughness, the thruster force profiles during Experiments 1 and Experiments 2 are shown in Figure 21 and Figure 22, respectively. Here, the results confirmed that the control part successfully generated the velocity set point x and z whenever necessary on the basis of the reference distance.

### 4.3. Comment

This work aimed at the design and development of an autonomous fish net pens tracking system. The obtained results are promising and indicate the capability of the schemes for efficiently detecting, localizing, and tracking the fish net pens in the real environment. However, the robustness of the proposed methods has to be routinely tested with a sequence of sea trials over time.

## 5. Conclusions

In this paper, a vision-based positioning and control scheme is described that can be used for auto-inspection of the fish net pens in underwater fish farms. We employed both stereo and monocular image design approaches for the input data acquisition and the traditional computer vision methods for the target position detection. The vision algorithm was integrated with a control module that allows the vehicle to perform the traversing along the net plane. The system was tested both in a simulation and in a real environment. In terms of performance, we found that the monocular image-based method is more suitable than the stereo-image-based method. From the obtained results, we learned that the stereo-image-based method is highly influenced by the choice of hardware design, features extraction and computation cost. In contrast, the monocular image-based method is found to be easily adopted in real applications because of fewer requirements and lower computation costs. The scheme also avoids tracking the image features and does not suffer from repeated scenes in the input data. In the future, we are interested to overcome the existing limitations by performing more experiments, providing the state’s data, and evaluating the results with the true positions and state data. We also plan to modify the control part and integrate it with the feedback control law to make the scheme more automatic.

## Figures and Tables

**Table 1:** Review of fish net tracking and inspection techniques.

Ref.	Technique	Task	Remarks
[4]	Bilateral filter	Damage detection	Does not incorporate the vehicle control
[5]	Kalman filter	Structure detection	Positions data is not communicated
[6]	Fourier Transform	Pose estimation	Does not incorporate the vehicle control
[7]	Canny edge detector	Hole detection	Does not perform top-down tracking
[8]	Deep learning	Damage detection	Experience pose estimation error
[11]	DVL	Net inspection	Not robust to noise
[12]	Hough transform	Damage detection and water quality monitoring	The vehicle is controlled manually
[13]	IoT network	Water quality monitoring	Does not consider net tracking
[14]	Canny edge detector	Net status inspection	Required predetermined target location on the net plane
[15]	Canny edge detector	Net status inspection	Does not consider vehicle control
[17]	Hough transform	Hole detection	Worked on offline images without control system
[21]	Canny edge detector	Net status inspection	Not robust to sunlight and blurred images

## Data Availability

Not applicable.
